# Three Years with the Army of the Potomac—A Personal Military History

**Published:** 1912-11

**Authors:** William Warren Potter

**Affiliations:** Buffalo, N. Y., Brevet Lieutenant Colonel, U. S. Volunteers; Surgeon in Charge of First Division Field Hospital Second Army Corps; Surgeon 57th Regiment New York Volunteers; Assistant Surgeon 49th Regiment New York Volunteers; Recorder Second Division Hospital Sixth Army Corps, etc., etc.


### Three Years with the Army of the Potomac—A Personal Military History.

By William Warren Potter, M.D., Buffalo, N. Y., Brevet Lieutenant Colonel, U. S. Volunteers; Surgeon in Charge of First Division Field Hospital Second Army Corps; Surgeon 57th Regiment New York Volunteers; Assistant Surgeon 49th Regiment New York Volunteers; Recorder Second Division Hospital Sixth Army Corps, etc., etc.

*Reams' Station.*

Sunday, August 28. At the Burchard House. We returned here yesterday morning, after a week's absence at Reams' Station and in that direction. After seeing Warren firmly established in his grip on the Weldon R. R., the Second Corps was set to work, tearing up the road. On Thursday, the 25th, after having destroyed about ten miles of the road, we reached Reams' Station, or rather we had already destroyed the track two or three miles farther on, when the enemy attacked us in considerable force. Several different assaults were made, five in all, when we were finally driven out. It pains me to say that the Second Corps, though for the first time in its history, behaved disgracefully, some of the men running like sheep. Hancock and his entire staff strove in vain to rally them, exposing themselves to a terrible fire in so doing; even the Medical Director, Commissary of Subsistence, and the Chief Quartermaster were called upon to perform the ordinary duties of aides, which they all did in a gallant manner. But nothing could be done to retrieve the lost fortunes, and with the coming on of darkness we made preparations to retreat within the Petersburg lines. Captain E. P. Brownson, the Corps mustering officer, was killed, and Lieutenant Colonel Francis A. Walker, Assistant Adjutant General, was captured. These were the only casualties to the Corps Staff. Walker was sent with an order to Miles, commanding the First Division, and unconsciously rode into the the enemy' lines through a gap in our own. Colonel Beaver received a wound in his right thigh that necessitated its amputation through its upper third, which Dr. Wishart and I performed. The Colonel was just returning from absence by reason of his Petersburg wound, having arrived only that day, and was going out on foot to take command of the Fourth Brigade when he was struck by a rifle ball that shattered his thigh. I had him brought hither from the field on a stretcher, by a detail of sixteen men, distance about ten miles, and he is now inside the Burchard Mansion doing well, and has, I think, a fair prospect for recovery.

Our loss in killed and wounded was not large, but many were taken prisoners, and our military prestige is gone, though, let it be hoped, only temporarily. We lost nine guns, including McKnight's 27th N. Y. Battery in which Lieutenant W. S. Bull is serving. This is the second time McKnight has come to grief during the campaign. On June 22 he lost four guns; but, when, at Deep Bottom in July, the First Division took four thirty-pounders back again, it was regarded by the boys as pretty good interest for the brief loan of McKnight's Napoleons. After all we have passed through during the past week, it seems good to get back here to the Burchard house. Last night the firing continued almost the whole night, and I amused myself watching the shells as they sailed through the air, the spectacle resembling a display of fireworks.

Tuesday, August 30. Heavy firing was kept up until a late hour last night, but it amounted to little or nothing in its effects. Colonel Beaver is still doing well, though not out of danger yet; he bears his suffering bravely, however, and keeps up good courage, which is half the battle. I hear that Colonel Brown, missing since May 12, has been exchanged and is at his home in Erie, Pa. Miss Gilson paid me a call this afternoon, but she did not dismount as she was in some haste to return to City Point. The weather is delightful now, not as hot as it has been, and everybody feels more cheerful. General Hancock comes frequently to see Colonel Beaver, and cheers him by saying, nearly every time, "Never mind the leg, Beaver, you'll be a Brigadier General now." I took a ride in the country yesterday, and called at a farm house where they served watermelons and peaches in bountiful quantities, so that my appetitie in that direction was appeased for the nonce.

Friday, September 2. We are still at the Burchard house which, taken altogether, is the best location for a hospital I have yet seen within all the Petersburg lines. It is near enough to the line of entrenchments to be within easy reach, and far enough away to be out of immediate danger; it is off the main thoroughfare sufficiently far to escape much of the dust, and the grounds are well adapted to the proper arrangement of the hospital tents, and to ensure good drainage. The tents are pitched in the peach orchard adjoining the kitchen garden, which latter is well fenced —the only fence anywhere in sight. The family is entirely dependent upon the commissariat of the hospital for daily subsistence, and in return, Mrs. Burchard gives me such unrestricted use of the house as I may demand. Thus far I have only asked for quarters for Colonel Beaver inside the house, who is occupying the back parlor, looking out upon the garden over a veranda.

I have built a fine brick bake-oven near one of the out buildings, that supplies us with fresh bread, pies and other luxuries. Colonel Beaver seems to be doing well, although I regard his case as still dangerous. Mr. McCallister, his law partner from Bellfonte, Pa., arrived late last night, and will remain with the Colonel for a day or two. Miss Gilson is here taking care of him, and he seems more cheerful under her considerate and tender ministration. The weather is now delightful, and the nights cool enough for one to sleep well. We hear that McClellan has been nominated for President by the Democrats at Chicago. It is to be regretted that so good a soldier should be so poor a politician —and that on the wrong side. Dr. Norris has returned from his twenty days leave, having visited Niagara Falls during his absence.

Monday, September 5. I have suffered from neuralgia in my face for a day or two, probably due to an imperfect tooth, which is the first illness I have had for more than a year. Dr. Wishart has extracted the offending tooth, and I have finally obtained relief. Today has been warmer than any day since September came in, the weather of late having been quite fall-like in character. Colonel Beaver seems better today, and I have strong hopes of his recovery. Miss Gilson is still here rendering great service to the Colonel, frequently entertaining him, and the rest of us, I might say as well, with music, both vocal and instrumental, as she sings and plays beautifully. She is a niece of the Hon. Mr. Fay, of Chelsea, Mass., who is a prominent member of the U. S. Sanitary Commission. Mr. Fay, himself, pays frequent visits to the hospital, and has made many friends among the patients, as well as the staff, who all like him very much indeed. He comes up from City Point almost every day while his niece is with us, and takes the tenderest care of her in every way. Colonel Beaver gave me his photograph today, which I sent home at once, and it is now in my military album.

Last night, at midnight, General Grant ordered every gun bearing on the enemy's line, to fire a shotted national salute (34 guns) in celebration of Sherman's capture of Atlanta. It was a magnificient spectacle, and had not continued long ere the enemy replied with considerable vigor, the fusilade lasting about two hours. A fifteen inch mortar near the Appomattox on Butler's front, called "The Swamp Angel," was particularly noticeable for its share of the work. It can be loaded and fired but four times an hour, owing to the fact that it requires nearly fifteen minutes to clean it after each discharge. The fuse, as it goes upwards into the heavens looks like a star, particularly while it poises for a moment, before descending into the enemy's lines. The air seemed full of shells for a time, and it was a grand pyrotechnic display. Some of the shells burst in the vicinity of the hospital, but did no harm.

A cattle raid has been made in the vicinity of the hospital on the Army herd, the whole of which, 2,500 head, was captured and driven off by the enemy, not more than 100 stragglers having been found in the woods afterwards. I am told that the scheme was planned and executed by General Dearing, who knew every bridle path in this region where he courted his wife, having, no doubt, rambled over the country many times with her during the happy courtship. The cattle were herded about a mile in rear of the hospital, and when I heard the firing so distinctly as I did, and early in the morning, too, I at first thought it something more serious; as it was, however, it simply turned out a clever piece of theft, which every one must concede. It temporarily embarrassed the Army for beef, but this was overcome in a few days; while to the enemy it was of inestimable value just at this time, when the Rebel Commissariat is at low tide.

Saturady, September 10. Burchard House. We have moved twice during the week, once away from here, and once back again. Col. Morgan, Chief of Staff, (Second Corps) told Dr. Dougherty that the enemy was expected in our rear on the Prince George Road, and the doctor gave us an order on Tuesday, about five o'clock P. M., to move immediately. We obeyed the order promptly, although it was in the midst of a driving rain, going out towards the front, where we had our hospital about 200 yards in rear of the line of battle of the 2d Division for forty-eight hours. The scare passed over at the end of that time, and we are now back again as good as before.

I have been to City Point today where I ordered a bill of goods, through the Medical Purveyor, from Baltimore for the hospital, amounting to $1,200.00. This will give the patients canned fruits, oranges, lemons and many other luxuries not furnished by the Commissary Department, which will be paid for out of the hospital fund. This fund accrues from the savings in the rations on the march and in an active campaign, when we cannot use all we are entitled to; such as are not drawn each day are passed to the hospital as a cash credit, and against this fund we can draw for the purchase of whatever we need that is not furnished in the supply tables of the several Departments, Medical, Commissary, or Quartermaster.

I am beginning to make preparations to leave the service, though I shall not probably be mustered out until the 23d. Lieutenant Snyder, Q. M. 57th, will go at the same time. Dr. Dougherty is very anxious for me to say that I will come back after a month spent at home, and take charge of the hospital again, referring to the subject in some way almost every time I see him. Colonel Beaver is improving day by day, and I now have strong hopes of his recovery. Indeed, there is little doubt on the subject. Miss Gilson has returned to City Point, and we were all sorry to lose her excellent services, as well as part with her cheerful company. I saw Lieutenant Bull yesterday, who informs me that he expects to go to Buffalo on recruiting service in a few days; so we shall probably meet there before long.

Wednesday, September 14. I have just been informed that I can be mustered out tomorrow if I desire, that being the termination of three years' service in my case, the date of my commission being September 15, 1861. In case I accept discharge tomorrow I shall start for home next Monday, the 19th. It will take some time to settle my accounts with the Government, and to transport my horses, so I shall be kept in Washington for a few days, and shall not reach home probably until the 25th, or thereabouts. The weather has been cooler again for a few days past, so much so that overcoats have been comfortable morning and evening. Miss Gilson paid me a visit yesterday, remaining to dinner. Colonel Beaver is continuing to improve and I am confident he will recover.

The rebels began a furious cannonade on our front about two hours ago, that has not yet ceased though it seems to be dying down somewhat. Some of the shells burst over Army Headquarters, which are located a short distance to our front. Last evening Dr. McParlin, Medical Director of the Army, and Colonel Wilson, Chief Commissary A. of P., came over on foot and inspected the hospital, after which Colonel Wilson gave us some fine music on the piano in Mrs Burchard's parlor.

*Mustered Out.*

Thursday, September 15, 1864. Today being the completion of three years' service with the Army of the Potomac, and the end of the term for which I originally entered service, I have been duly discharged from the military service of the United States. I have transferred the hospital to Dr. James E. Pomfret, Surgeon 7th N. Y. H'y Art, who receipts to me for all the property, and for which invoices are now being made out.

Dr. Pomfret remained in charge until the following February, when he was succeeded by Dr. Charles S. Hoyt, Surgeon 39th N. Y., Executive Officer of the Hospital. Dr. Hoyt held the position until the end of the war, and is at present Secretary of the State Board of Charities.

In concluding this narrative it may be interesting to note: that no corps of the Army had a greater aggregate of casualties than the Second, and that from May 3d, the date of the opening of the campaign, to September 19th, the day of my departure, the register showed that we had treated 8,000 patients in the First Division Hospital, of which number over 6,000 had been wounded.

